# Navigating Misplaced Leads: A Case of Left Ventricle (LV) Lead Placement via a Patent Foramen Ovale (PFO) in a Dual-Chamber Permanent Pacemaker (PPM)

**DOI:** 10.7759/cureus.82835

**Published:** 2025-04-23

**Authors:** Jhiamluka Solano, Dalia Admed, Aia Ahmed, Shuja Abbas, Adnan Ahmed, Renjith Antony

**Affiliations:** 1 Resident Doctor Committee, Royal College of Physicians, London, GBR; 2 Education Committee, Academy of Medical Educators, Cardiff, GBR; 3 Cardiology, Scunthorpe General Hospital, Scunthorpe, GBR; 4 Cardiology, Hull University Teaching Hospital NHS Trust, Hull, GBR; 5 Cardiology, Hull University Teaching Hospitals NHS Trust, Hull, GBR; 6 Cardiology, Hull University Hospital, Castle Hill Hospital, Hull, GBR; 7 Cardiology, Hull Univeristy Teaching Hospital, Hull, GBR

**Keywords:** lead misplacement, left ventricle, mitral regurgitation, pacemaker, patent foramen ovale

## Abstract

Pacemaker lead misplacement is a rare but clinically significant complication of pacemaker implantation. We present the case of a male patient in his 80s with a permanent pacemaker (PPM) who was found to have an inadvertently misplaced right ventricular (RV) lead coursing through a patent foramen ovale (PFO) into the left ventricle (LV). The patient presented with progressive dyspnoea and was found to have moderate mitral regurgitation related to lead interference with valve closure. Imaging modalities confirmed the lead's anomalous position. A multidisciplinary discussion concluded that conservative management with anticoagulation was the safest approach. This case highlights the importance of multimodal imaging in identifying lead misplacement and the clinical implications of inadvertent LV pacing. Furthermore, it underscores the importance of routine post-implantation assessment to detect lead displacement, which can often occur without symptoms. Early identification through systematic evaluation is essential to prevent complications and ensure optimal device function.

## Introduction

Pacemaker lead misplacement is a rare but significant complication of permanent pacemaker (PPM) implants, present in 0.3% of cases, often occurring when the lead is unintentionally positioned in the left side of the heart, such as the left atrium or left ventricle (LV) [1, 2]. This can happen in patients with an interatrial septal defect, like a patent foramen ovale (PFO), where the lead crosses from the right side of the heart to the left [3]. Misplacement into the LV can lead to a range of complications, including thromboembolism, arrhythmias, and mitral regurgitation, due to lead-induced tethering of the mitral valve [4]. Diagnosis typically requires multimodal imaging, including echocardiography, CT, and fluoroscopy [5]. This case presents an elderly patient with an inadvertently misplaced pacemaker lead in the LV, highlighting the clinical challenges and management considerations associated with this complication.

## Case presentation

**Figure 1:**
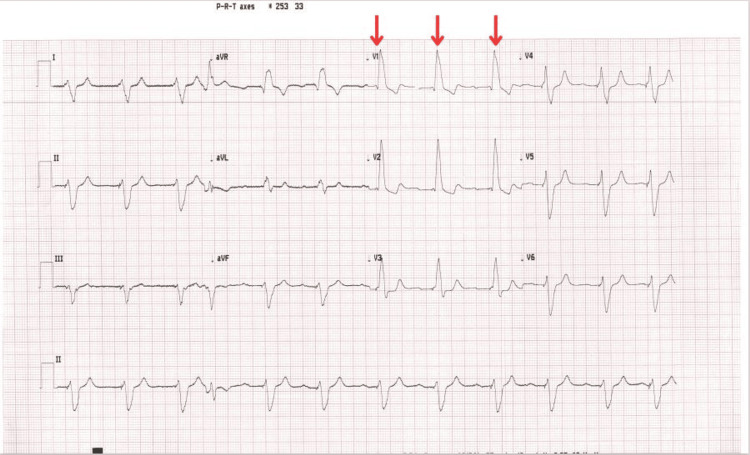
The patient's ECG on admission Arrows show the QRS right bundle morphology in lead V1.

**Figure 2:**
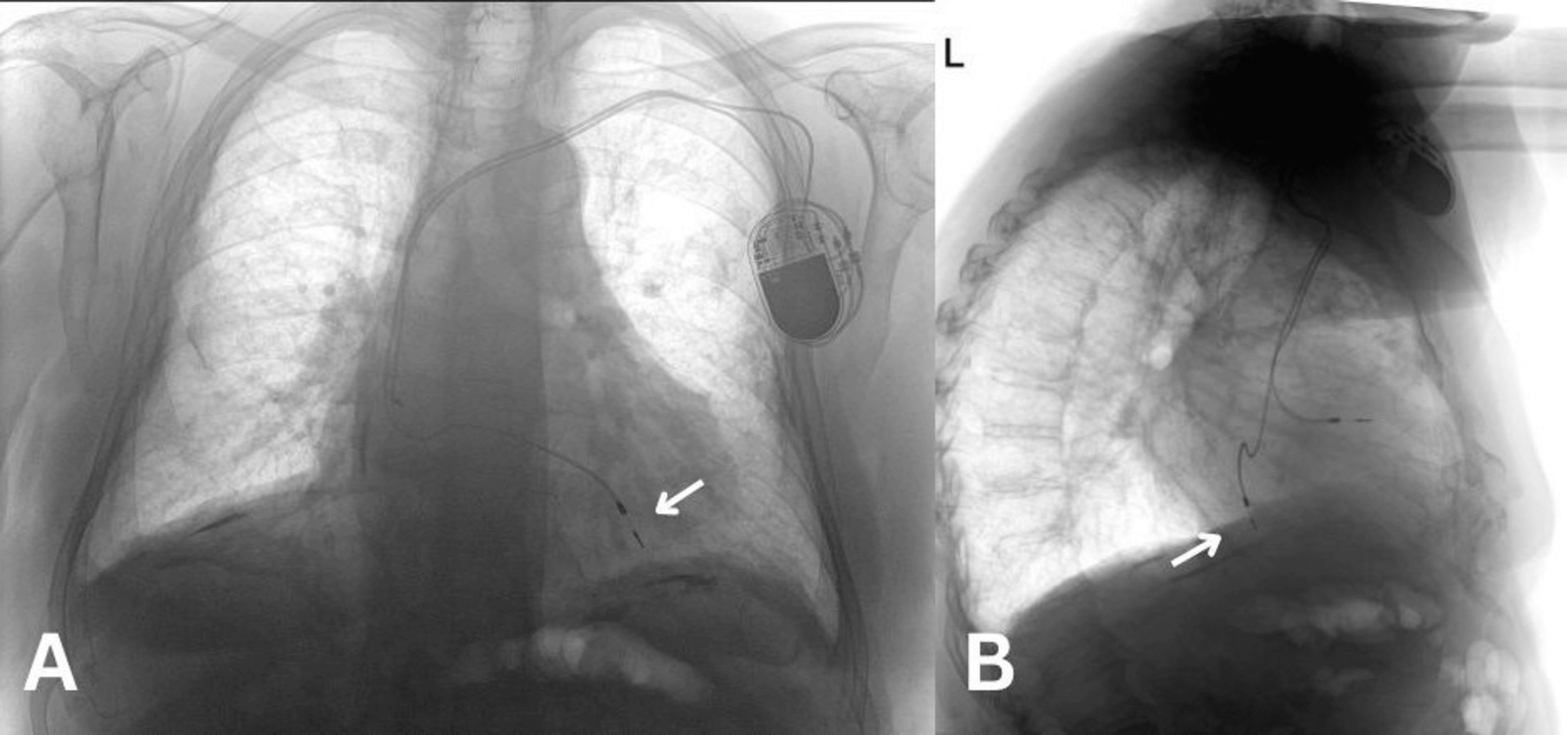
Chest X-ray in the anteroposterior (A) and lateral view (B) The arrow shows the right ventricular lead in the left ventricle.

A male patient in his early 80s, with a history of complete heart block requiring pacemaker implantation 12 years prior, presented with progressive dyspnoea classified as New York Heart Association (NYHA) class II. Clinical examination was largely unremarkable except for a soft systolic murmur at the mitral area. Electrocardiogram (ECG) demonstrated a paced rhythm with a right bundle branch block (RBBB) morphology, suggestive of LV pacing (Figure 1), and a chest X-ray (CXR) in the anteroposterior (AP) view (Figure 2A) showed an abnormal superior course of the ventricular lead toward the left atrium and LV, and the lateral view (Figure 2B) revealed a posteriorly positioned ventricular lead, consistent with LV lead misplacement.

**Figure 3:**
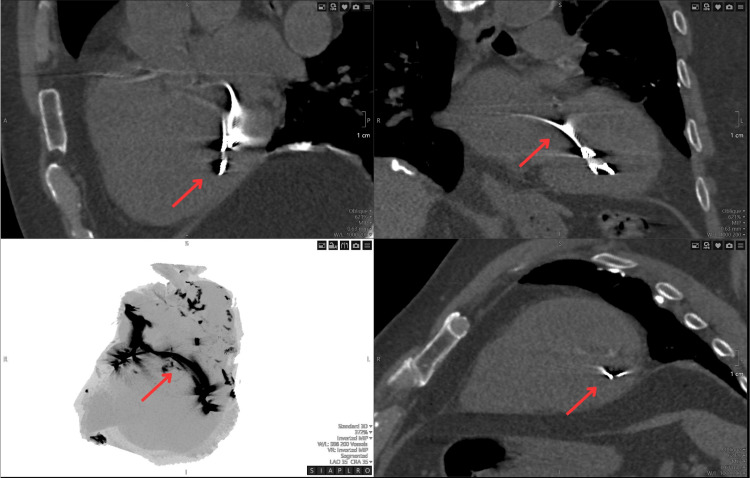
Computed tomography (CT) of the thorax Multiplanar reformation or reconstruction (MPR) oblique views with a focus on bringing the left ventricle (LV) lead in view.

The transthoracic echocardiography (TTE) identified the pacemaker lead traversing a PFO into the LV via the left atrium and mitral valve, with moderate mitral regurgitation due to lead-induced tethering of the posterior mitral leaflet (Video 1). The computed tomography (CT) of the thorax, as confirmed on a retrospective review, showed the LV lead misplacement (Figure 3).

**Video 1:** Transthoracic echocardiography (TTE) A: Three-chamber view with lead crossing the mitral valve (arrows); B: Three-chamber view with colour Doppler; C: Four-chamber view; D: Four-chamber view with colour Doppler showing mitral regurgitation (arrows).

The patient was discussed in a multidisciplinary heart team meeting. Given the chronicity of lead positioning, the risk of systemic embolisation, and the presence of moderate mitral regurgitation, the decision was made to manage conservatively. The patient was started on long-term anticoagulation therapy with a direct oral anticoagulant (DOAC) to mitigate thromboembolic risk due to the patient's preference. Lead repositioning was deemed high-risk due to the potential complications of chronic lead extraction. He was subsequently followed up in both cardiology and pacing clinics, where he remained stable.

## Discussion

This case illustrates an uncommon complication of pacemaker implantation, in which an RV lead was inadvertently positioned in the LV via a PFO, a phenomenon previously reported in the literature [6]. The ECG evaluation may reveal atypical findings, such as an RBBB-like morphology in cases of RV lead displacement or unintentional LV pacing, which should prompt further diagnostic assessment, as usually in RV pacing, you expect to see a left bundle branch block (LBBB) morphology [7]. In this context, routine post-implantation CXR, primarily performed to exclude pneumothorax, can provide critical anatomical information. Abnormal lead positioning identified on CXR should raise suspicion for malposition and lead to additional imaging, as early detection and correction are essential to mitigate the risk of adverse outcomes.

Pacemaker lead misplacement into the LV has been reported to occur through various mechanisms, including direct perforation, interatrial septal defects, or PFOs [4]. A misplaced LV lead is particularly concerning due to its potential to cause systemic embolisation, left ventricular dysfunction, and valvular impairment [6]. In our patient, the lead caused moderate mitral regurgitation by tethering the posterior mitral leaflet, which interfered with its normal closure [8].

Systemic thromboembolic events are a significant risk in patients with an LV-paced lead. The left-sided circulation increases the likelihood of embolic stroke, which is why anticoagulation is strongly recommended in such cases [9,10]. Although lead repositioning may seem like a definitive solution, it is associated with significant procedural risks, particularly in elderly patients or those with chronic lead placement, like our case [6]. The risk of lead extraction includes cardiac perforation, tamponade, and vascular injury, which can be life-threatening [2]. For this reason, conservative management with long-term anticoagulation is often preferred in stable patients [9].

Multimodal imaging plays a critical role in diagnosing this complication. While ECG patterns such as RBBB morphology can hint at LV pacing, ECG remains the primary diagnostic tool for confirming lead misplacement [11]. Additional imaging, including CT and fluoroscopy, can further delineate the course of the lead and confirm its location [5]. Our case highlights how retrospective review of previously obtained imaging, such as CT, can assist in identifying lead malposition that was not initially suspected.

Given the increasing number of pacemaker implantations worldwide, recognising lead misplacement is crucial for preventing complications and optimising patient outcomes [12-14]. A high index of suspicion should be maintained in patients with unexplained dyspnoea, abnormal pacing morphologies, or new valvular dysfunction following device implantation [2]. Current advancements in leadless pacing technologies and imaging modalities may reduce the incidence of such complications and improve patient safety.

## Conclusions

This case underscores the critical role of multimodal imaging in diagnosing pacemaker lead misplacement and guiding management decisions. In patients with LV pacing, a high index of suspicion and thorough evaluation are essential to prevent potential complications and optimise patient outcomes. Conservative management with anticoagulation can be a safe alternative to lead repositioning in select patients, particularly when procedural risks are high.
